# Protective Effect of Leaf Essential Oil from *Cinnamomum osmophloeum* Kanehira on Endotoxin-Induced Intestinal Injury in Mice Associated with Suppressed Local Expression of Molecules in the Signaling Pathways of TLR4 and NLRP3

**DOI:** 10.1371/journal.pone.0120700

**Published:** 2015-03-20

**Authors:** Shih-Chieh Lee, Jie-Sheng Hsu, Chien-Chun Li, Ke-Ming Chen, Cheng-Tzu Liu

**Affiliations:** 1 Department of BioIndustry Technology, Da-Yeh University, No. 168, University Rd., Dacun, Changhua, Taiwan, Republic of China; 2 School of Nutrition, Chung Shan Medical University, No. 110, Sec. 1, Chien Kuo N. Rd., Taichung, Taiwan, Republic of China; 3 Department of Parasitology, Chung Shan Medical University, No. 110, Sec. 1, Chien Kuo N. Rd., Taichung, Taiwan, Republic of China; 4 Department of Nutrition, Chung Shan Medical University Hospital, No. 110, Sec. 1, Chien Kuo N. Rd., Taichung, Taiwan, Republic of China; Chang-Gung University, TAIWAN

## Abstract

Endotoxin is a potent microbial mediator implicated in sepsis. We investigated the anti-inflammatory effect of leaf essential oil from *Cinnamomum osmophloeum* Kanehira (CO) of the linalool chemotype on endotoxin-injected mice. Mice were administered CO or vehicle by gavage before endotoxin injection and were killed 12 h after injection. Neither growth nor the organ weight or tissue weight to body weight ratio was affected by CO treatment. CO significantly lowered peripheral levels of tumor necrosis factor-α, interleukin (IL)-1β, IL-18, interferon-γ, and nitric oxide and inhibited the expression of toll-like receptor 4 (TLR4), myeloid differentiation primary response gene (88), myeloid differentiation factor 2, apoptosis-associated speck-like protein containing a caspase-recruitment domain (ASC), caspase-1, and Nod-like receptor family, pyrin domain containing 3 (NLRP3). CO also inhibited the activation of nuclear factor-ĸB, inhibited the activity of caspase-1 in small intestine, and ameliorated intestinal edema. Our data provide strong evidence for a protective effect of CO of the linalool chemotype in the endotoxin-induced systemic inflammatory response in close association with suppression of the TLR4 and NLRP3 signaling pathways in intestine.

## Introduction

Sepsis is a systemic inflammatory condition with the presence of infection that can lead to death of critically ill patients, mainly as the result of progression to multiple organ failure. Even though much effort has been made to understand the complex mechanism of sepsis to improve patient care, mortality from sepsis remains high [1]. This clinical condition is now recognized to result from the immunopathology initiated by the inflammatory response to the insult, which is followed by multiple tissue and organ damage mediated by excessive generation of proinflammatory cytokines [2]. In the exploration of the sepsis-associated immune response, endotoxin has raised the most interest because this lipopolysaccharide constituent of the cell wall of gram-negative bacteria has been recognized as the most potent microbial mediator in the pathogenesis of sepsis [3]. In experimental animals, administration of endotoxin induces systemic inflammatory response syndrome (SIRS) along with injury to multiple organs that is similar to the features of SIRS in patients. Thus, this experimental animal model is a common tool for investigation of therapeutic measures for sepsis [4].

Current understanding of the tissue damage in endotoxin-induced SIRS has proposed two signaling pathways of the innate immune system as possible targets for intervention, namely, toll-like receptor 4 (TLR4) and nod-like receptor family, pyrin domain containing 3 (NLRP3). It is well established that the receptor CD14/TLR4/myeloid differentiation protein 2 (MD2) in host cells, chiefly in cells of the innate immune system, is the major receptor activated by endotoxin [5]. Upon interacting with endotoxin, this TLR4 complex recruits and ligates with its adaptor protein, myeloid differentiation primary response gene (88) (MyD88), followed by the activation of certain transcription factors such as nuclear factor (NF)-κB for the expression of several genes. Activation of NF-κB eventually results in the generation of nitric oxide and the secretion of various proinflammatory mediators, of which tumor necrosis factor (TNF)-α and interleukin (IL)-1β are the mostly studied. Many researchers have proposed TNF-α and IL-1β to be the most effective pathogenic factors that eventually induce the development of organ failure [6, 7]. Recent study revealed that it is the transcription of pro-IL-1β but not IL-1β that is activated by NF-κB and that this occurs concomitantly with transcription of pro-IL-18. Study suggests that is necessary for these two cytokine progenitors to be cleaved by the converting enzyme caspase-1 before the cells can produce and secrete biologically active IL-1β and IL-18, respectively [8].

Upon activation, NLRP3 assembles with the apoptosis-associated speck-like protein containing a CARD (ASC) and the cytoplasmic enzyme caspase-1 to form a complex named the NLRP3 inflammasome. In general, caspase-1 presents in cells in an inactive form, and its activation is mediated by the NLRP3 inflammasome [8]. This inflammasome is therefore responsible for the production of IL-1β and IL-18 [8]. Although endotoxin is not a ligand for NLRP3, evidence has been shown for the contribution of this cytosolic pattern recognition receptor on endotoxin-induced inflammation. Endogenous stress as a consequence of TLR4 activation can lead to the release of damage-associated molecular pattern molecules (DAMPs), such as uric acid, that are ligands of NLRP3 [9]. Furthermore, NF-κB can enhance the expression of NLRP3 and thus can further promote the outcome of NLRP3 inflammasome activity by DAMPs [10].

*Cinnamomum osmophloeum* cultivated in Taiwan has been classified into six chemotypes according to the dominant compounds in their leaf essential oil: cinnamaldehyde type, cinnamaldehyde/cinnamyl acetate type, cinnamyl acetate type, linalool type, camphor type, and mixed type according to gas chromatography−mass spectrometry (GC/MS) and cluster analyses of their leaf essential oils [11]. We previously demonstrated the chemical composition and the anti-diabetic action of the leaf essential oil of *Cinnamomum osmophloeum* Kanehira (CO) of the linalool chemotype, which is associated with suppressed peripheral and tissue levels of proinflammatory cytokines in rats [12]. Previous study by others also showed CO to possess great potential as an anti-inflammatory agent as shown by inhibition of production of proinflammatory mediators in endotoxin-induced models *in vitro* and *in vivo*, but none of these studies was carried out with CO of the linalool chemotype, instead, cinnamaldehyde chemotype was usually used and cinnamaldehyde is generally suggested to be an active compound in CO [13–18]. In addition, direct evidence for the protective effect of CO on inflammation-induced tissue damage is still lacking. Given that impaired intestinal barrier function under stress has clinical implications for nosocomial infection in critically ill patients [19], the aim of the present study was to provide evidence for the effect of CO on inflammatory injury to the intestine in an endotoxin-induced SIRS mouse model. Specifically, on the basis of previous understanding of the role of the TLR4 and NLRP3 signaling pathways in SIRS-associated liver injury [8, 20], the present study hypothesized that these signaling pathways are also closely associated with SIRS-associated intestinal damage. Consequently, the present study intended to investigate the anti-inflammatory effect of CO with special focus on the regulation of the TLR4 and NLRP3 signaling pathways in the intestine.

## Materials and Methods

### Chemicals and Reagents

Phenylmethylsulfonyl fluoride (PMSF) and the protease inhibitor cocktail were purchased from Roche (Indianapolis, IN); the mouse TNF-α enzyme-linked immunosorbent assay (ELISA) kit and the mouse interferon (IFN)-γ ELISA kit were purchased from eBioscience (San Diego, CA); the mouse IL-1β ELISA kit and the rabbit anticaspase-1 antibody were purchase from Invitrogen (Carlsbad, CA); the IL-18 ELISA kit was purchased from Uscn (Wuhan, China); the Nitrate/Nitrite Colorimetric Assay Kit, the xanthine oxidase assay kit, the nuclear extraction kit, and the NF-κB (p65) transcription factor assay kit were purchased from Cayman (Ann Arbor, MI); the mouse anti-β-actin antibody, the horseradish peroxidase (HRP)-conjugated goat anti-rabbit antibody, and the goat anti-mouse immunoglobulin G antibody were purchased from Merck Millipore (Darmstadt, Germany); the rabbit anti-TLR4 antibody, the rabbit anti-MD2 antibody, and the rabbit anti-MyD88 antibody were purchased from GeneTex (Irvine, CA); the rabbit anti-NLRP3 antibody and the rabbit anti-ACS antibody were purchased from Biorbyt (San Francisco, CA); the caspase-1 fluorometric assay kit was purchased from Biovision (Meylan, France); the Western Lightning Plus-ECL was purchased from PerkinElmer (Boston, MA); the Bio-Rad protein assay kit was purchased from Bio-Rad Laboratories (Richmond, CA); the endotoxin from *Salmonella typhimurium* and all other chemicals were purchased from Sigma Chemical Company (St. Louis, MO).

### Plant materials and preparation of essential oil

Fresh *C*. *osmophloeum* Kaneh. leaves were collected from a research farm at National Chiayi University, in Shekou, a central Taiwan county (latitude N23° 29′ 0.24″ E120° 33′ 23.6″). The land accessed is privately owned by National Chiayi University. *Cinnamomum osmophloeum* Kaneh. in this research farm, which is not a protected species, is for the research and teaching use only. The leaves were collected in May 2009 and were identified by Prof. Kuen-Yih Ho, Department of Forestry and Natural Resources, National Chiayi University. A voucher specimen (SK-CO-09-M-14) is deposited at the herbarium of the botanical garden of National Chiayi University, Chiayi, Taiwan. The leaves were subjected to hydrodistillation in an 80-L steam-distillator for 2 h, a pilot-scale-modified Clevenger-type apparatus, after which the oil contents were identified. Leaf essential oils were stored in airtight containers in the dark at −20°C prior to analysis by GC/MS. The leaf essential oil was analyzed by GC/MS to composed (in order of decreasing content %): linalool (40.24), transcinnamyl acetate (11.71), camphor (9.38), cinnamaldehyde (6.87), 3-phenyl-2-propenal (4.06), caryophyllene (2.65), coumarin (2.13), bornyl acetate (1.72), limonene (1.53), α-(+)-pinene (1.38), estragole (1.31), and caryophyllene oxide (1.00). These 12 compounds were identified to have content greater than 1%; combined, they constituted 83.99% of the leaf essential oils analyzed [12].

### Animals and Experimental Procedure

Ten-week-old male C57BL/6J mice were purchased from the National Animal Breeding and Research Center (Taipei, Taiwan). The animals were kept under a 12-h light-dark cycle at an ambient temperature of 23°C and were given free access to water and standard rodent feed (Rodent Diet 5001; Purina Mills, Richmond, IN). After acclimatization for 1 week, animals were randomly assigned to 5 groups and received by gavage leaf essential oil of *C*. *osmophloeum* (CO; 6.5, 13, or 26 mg/kg body weight) or the vehicle (corn oil; 4 mL/kg body weight) every other day for 2 weeks. According to our previous study, CO doses in this range have an anti-inflammatory effect on streptozotocin-injected animals [12]. Endotoxin from *Salmonella typhimurium* (10 mg/kg body weight) was injected intraperitoneally 15 days after the first administration of CO. The animals’ food supply was withdrawn followed by the injection. The control mice, which had received corn oil, were injected with the same volume of sterile saline. The mice were killed by carbon dioxide euthanasia 12 h after the injection. Blood was collected and the mesenteric lymph nodes (MLNs) and the intestine were removed immediately. MLNs and serum and plasma prepared freshly from blood samples were stored at −80°C until use within 1 week. Housing conditions and experimental procedures were performed according to the NIH *Guide for the Care and Use of Laboratory Animals*, and all protocols were approved by the ethical committee for animal experimentation of Chung Shan Medical University, Taichung, Taiwan.

### Preparation of Intestinal Tissue Samples

Immediately after the intestine was removed, the ileum segment (defined as the intestinal segment 10 cm proximal to the cecum) was irrigated with cold phosphate-buffered saline (pH 7.2) containing 1 mM phenylmethylsulfonyl fluoride to remove the intestinal contents. Mucosa was prepared as described previously [18] and was stored at −80°C for the analysis of xanthine oxidase, caspase-1, NF-κB, and Western blotting. Part of the segment was fixed in 10% neutral buffered formalin for histological analysis.

### Biochemical Analysis of Blood Samples

The concentration of TNF-α, IL-1β, IFN-γ, or IL-18 in peripheral blood was analyzed with the use of the ELISA kits of mouse TNF-α, IL-1β, IFN-γ, or IL-18, respectively. Nitrate/nitrite concentrations in blood samples were determined with the nitrate/nitrite colorimetric assay kit. The assay procedure was in accordance with the manufacturer’s instructions, and the results were analyzed with a microplate reader (VersaMax; Molecular Devices Ltd., Sunnyvale, CA).

### Xanthine Oxidase and Caspase-1 Activity Assay

The activity of xanthine oxidase in the intestinal mucosa or the activity of caspase-1 in MLNs and in the intestinal mucosa was determined with the xanthine oxidase assay kit or the caspase-1 fluorometric assay kit, respectively, in accordance with the manufacturer’s instructions. The fluorescence intensity of the product generated from the activity of xanthine oxidase or from caspase-1 was analyzed by using excitation at 520–550 nm and emission at 585–595 nm or excitation at 400 nm and emission at 505 nm, respectively, with a FlexStation 3 Microplate Reader (Molecular Devices, Silicon Valley, CA). The resultant xanthine oxidase activity detected was calculated by using a standard curve and expressed as μU/mg protein. The resultant activity of caspase-1 detected was expressed as percentage of the control.

### Nuclear Extraction and NF-kB Determination

NF-κB typically resides in the cytoplasm of cells as a complex with members of the IκB inhibitor family of proteins and translocates into the nucleus upon stimulation, such as by endotoxin and certain proinflammatory cytokines [21]. Consequently, the detection of NF-κB in the nucleus represents the activation of this transcription factor. The present study extracted nuclei from the MLNs and ileum mucosa with a nuclear extraction kit according to the manufacturer’s instruction. The nuclear extracts were subsequently determined for the content of NF-κB with the NF-κB (p65) transcription factor assay kit. The assay procedure was in accordance with the manufacturer’s instructions, and the results were analyzed with a microplate reader (VersaMax; Molecular Devices Ltd., Sunnyvale, CA).

### Western Blotting of MLNs and Ileum Mucosa

MLNs and ileum mucosa were homogenized in radioimmunoprecipitation assay buffer (50 mM Tris-HCl pH 7.4, 150 mM NaCl, 1% Triton X-100, 0.25% deoxycholate) supplemented with a protease inhibitor cocktail. The resultant supernatants were subjected to 4–12% Bis-Tris gel electrophoresis at 20 μg of protein per lane. The proteins were electroporated onto PVDF membranes and immunoblotted with anti-TLR4 antibody (1:500), anti-MD2 antibody (1:2000), anti-MyD88 antibody (1:1000), anti-NLRP3 antibody (1:500), anti-ACS antibody (1:1000), and anticaspase-1 antibody (1:1000). β-Actin was used as an internal control and was blotted with anti-β-actin antibody at a 1:5000 dilution. HRP-conjugated anti-rabbit or anti-mouse immunoglobulin G antibody was used at a 1:1000 dilution as the second antibody. Antibody-bound protein on the PVDF membrane was visualized by using an enhanced chemiluminescence substrate, Western Lightning Plus-ECL, according to the manufacturer’s specifications. Films were scanned by using a Luminescent Image Analyzer (FUJIFILM LAS-1000; FujiFilm, Tokyo, Japan). For quantification, the band intensities were analyzed with NIH Image software and were expressed as fold of relative intensity of that of the control. Protein assays were performed by using Bio-Rad protein assay kits.

### Histologic Analysis of Intestinal Integrity

The distal ileum fixed in 10% neutral buffered formalin was embedded in paraffin, sectioned at 5 μm, and stained with hematoxylin and eosin to evaluate the destruction of the villus architecture of the mucosa.

### Statistical Analysis

The data are expressed as the mean ± SD and were analyzed by one-way analysis of variance. Student’s t-test was used to detect differences in means between the control group and the endotoxin-injected mice. Duncan’s multiple-comparison test was used to detect differences among the means of the endotoxin-injected groups. P values of <0.05 were considered to be significant. All statistical analyses were performed with commercially available software (SPSS 12 for Windows; SPSS Inc., Chicago, IL).

## Results

### Body Weight and Organ Weight-to-Body Weight Ratio

Mice were pretreated with CO for 2 weeks before the induction of SIRS with endotoxin. During the pretreatment period, the body weight gain of the mice that received the vehicle was 0.53 ± 0.21 g, which was similar to that of the mice pretreated with the low, medium, or high dose of CO (0.62±0.97 g, 0.52±0.65 g, and 0.98±0.99 g, respectively). Thus, the tested doses of CO did not significantly affect the growth of these animals. After the induction of SIRS, organs were collected and the organ weight-to-body weight ratio was calculated as shown in Table 1. SIRS was associated with a significant elevation of the ratios of spleen weight and MLN weight to body weight. Administration of CO did not significantly affect the organ weight-to-body weight ratio in endotoxin-induced SIRS mice (Table 1).

**Table 1:** Tissue or Organ Weight to Body Weight Ratio of Control Mice or Endotoxin-Injected Mice That Did or Did Not Receive CO^a^.

organ wt/body wt x 100 (%)	control-V	endotoxin-V	endotoxin-COL	endotoxin-COM	endotoxin-COH
**spleen**	0.22 ± 0.02	0.33 ± 0.04^#^	0.31 ± 0.03	0.31 ± 0.02	0.32 ± 0.05
**kidney**	0.65 ± 0.06	0.71 ± 0.07	0.68 ± 0.07	0.69 ± 0.05	0.72 ± 0.06
**liver**	4.51 ± 0.61	4.80 ± 0.40	4.85 ± 0.34	4.96 ± 0.30	4.29 ± 1.52
**mesenteric lymph nodes**	0.07 ± 0.01	0.11 ± 0.01^#^	0.10 ± 0.02	0.10 ± 0.02	0.10 ± 0.03

^a^Values are the mean ± SD for six mice per group.

control-V, control mice treated with vehicle; endotoxin-V, endotoxin-injected mice treated with vehicle; endotoxin-COL, endotoxin-injected mice treated with 6.5 mg/(kg bw) of CO; endotoxin-COM, endotoxin-injected mice treated with 13 mg/(kg bw) of CO; endotoxin-COH, endotoxin-injected mice treated with 26 mg/(kg bw) of CO.

#Significantly different from the control group (p < 0.05).

### Peripheral Levels of Nitrate/Nitrite and Proinflammatory Cytokines

SIRS induced a significant elevation of several proinflammatory cytokines, including IL-1β, TNF-α, IFN-γ, and IL-18, in peripheral blood as expected (Table 2). Compared with the vehicle-treated mice, the mice pretreated with CO had significantly lower levels of IFN-γ and IL-18 at all tested doses (p<0.05) and significantly lower levels of IL-1β and TNF-α at doses of 13 and 26 mg/kg CO (p<0.05). In addition, the inhibitory effect of CO was dose dependent. At the highest dose, the inhibitory effect of CO on IL-1β, TNF-α, IFN-γ, and IL-18 was up to 48.9%, 51.8%, 35.4%, and 67.7%, respectively. The peripheral level of nitrate/nitrite was also elevated by endotoxin as expected, whereas pretreatment with CO at as low a dose as 6.5 mg/kg body weight had a suppressive effect on this level (Table 2). The 6.5, 13, and 26 mg/kg doses of CO suppressed the level of nitrate/nitrite in mice up to 34.9%, 39.8%, and 49.4%, respectively (Table 2).

**Table 2:** Peripheral Concentration of Nitrate/nitrite and Proinflammatory Cytokines of Control Mice or Endotoxin-Injected Mice That Did or Did Not Receive CO^a^.

	control-V	endotoxin-V	endotoxin-COL	endotoxin-COM	endotoxin-COH
**nitrate/nitrite (nmol/mL)**	40.6 ±21.8	624.0 ± 96.9^#^ ^a^	406.3±137.5^b^	375.4 ±148.4^b^	315.6 ± 76.6^b^
**IL-1β (pg/mL)**	62.6 ± 29.2	215.3 ±44.4^#^ ^a^	181.3 ±42.8^a^	119.6 ± 35.3^b^	110.0 37.2^b^
**TNF-α (pg/mL)**	37.9 ± 10.5	474.5 ± 22.4^#^ ^a^	421.4 ± 61.9^a^	278. 5 ± 45.4^b^	228.6 40.6^b^
**IFN-γ (pg/mL)**	1.63 ± 0.51	106.34 ± 8.25^#^ ^a^	71.42 ± 6.19^b^	70.37 ± 2.39^b^	68.67 2.90^b^
**IL-18 (pg/mL)**	45.3 ± 11.7	283.0 ± 78.60^#^ ^a^	167.4 ± 65.3^b^	136.4 ± 47.5^bc^	91.5 22.7^c^

^a^Values are the mean ± SD for six mice per group.

control-V, control mice treated with vehicle; endotoxin-V, endotoxin-injected mice treated with vehicle; endotoxin-COL, endotoxin-injected mice treated with 6.5 mg/(kg bw) of CO; endotoxin-COM, endotoxin-injected mice treated with 13 mg/(kg bw) of CO; endotoxin-COH, endotoxin-injected mice treated with 26 mg/(kg bw) of CO.

#Significantly different from the control group (p < 0.05).

a, b, and c indicate the means within a row not sharing the same superscript letter are significantly different (p < 0.05).

### Expression of TLR4 Pathway-Related Molecules and NF-κB Activation in Ileum Mucosa and MLNs

**Fig 1:**
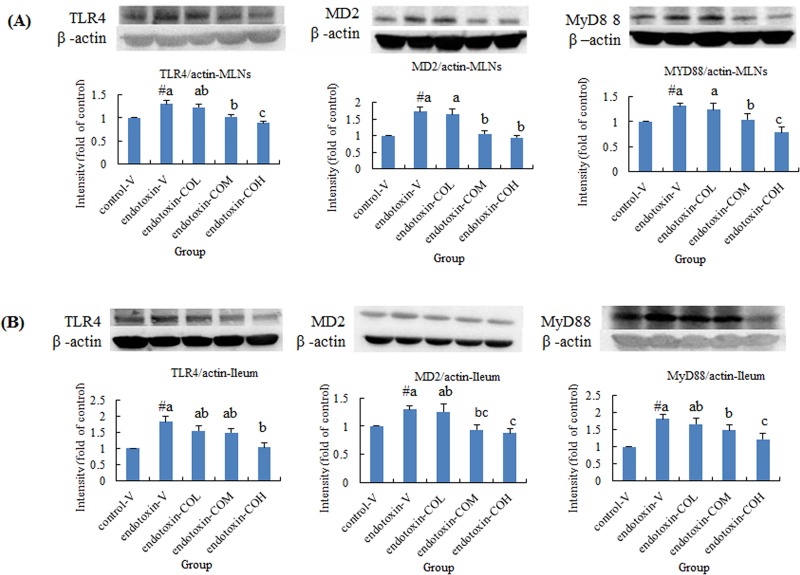
Effect of CO on the expression of TLR4, MD2, and MyD88 in mesenteric lymph nodes (A) and in ileum mucosa (B) of endotoxin-injected mice. Mice received by gavage 6.5 mg/kg CO (endotoxin-COL), 13 mg/kg CO (endotoxin-COM), 26 mg/kg CO (endotoxin-COH), or the vehicle (endotoxin-V) every other day 8 times followed by injection with endotoxin from *S*. *typhimurium* (i.p., 10 mg/kg body weight). Control mice were pretreated with vehicle (control-V) followed by injection with saline. Samples were collected at 12 h after injection. Data are means ± SDs for three mice in each group. #Significantly different from the control (p < 0.05). a, b, and c not sharing the same letter are significantly different (p < 0.05).

**Fig 2:**
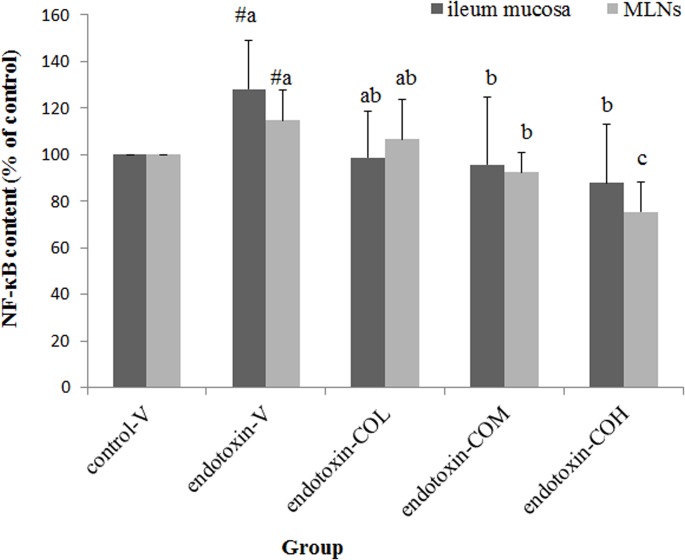
Effect of CO on NF-κB activation in ileum mucosa and in mesenteric lymph nodes of endotoxin-injected mice. Mice received by gavage 6.5 mg/kg CO (endotoxin-COL), 13 mg/kg CO (endotoxin-COM), 26 mg/kg CO (endotoxin-COH), or the vehicle (endotoxin-V) every other day 8 times followed by injection with endotoxin from *S*. *typhimurium* (i.p., 10 mg/kg body weight). Control mice were pretreated with vehicle (control-V) followed by injection with saline. Samples were collected at 12 h after injection. Data are means ± SDs for six mice in each group. #Significantly different from the control (p < 0.05). a, b, and c the same tissue not sharing the same letter are significantly different (p < 0.05).

Induction of SIRS with endotoxin not only increased the expression of TLR4, MD2, and MyD88 in the local lymph organ, namely MLNs, but also dramatically induced the expression of these molecules in ileum mucosa (Fig. 1A and B, respectively). In SIRS mice pretreated with CO, the expression of TLR4, MD2, and MyD88 in both tissues was significantly suppressed compared with that in the vehicle-treated mice, and this effect of CO was dose dependent (Fig. 1A and B). We next carried out further investigation of the nuclear content of the transcription factor NF-κB, which translocates from the cytoplasm to the nucleus upon activation. We found that SIRS-induced activation of NF-κB in both MLNs and ileum mucosa was reversed by pretreatment with CO in a dose-dependent manner with statistical significance at doses of 13 and 26 mg/kg (Fig. 2).

### Expression of NLRP3 Inflammasome-Related Molecules and Caspase-1 Activity in Ileum Mucosa and MLNs

**Fig 3:**
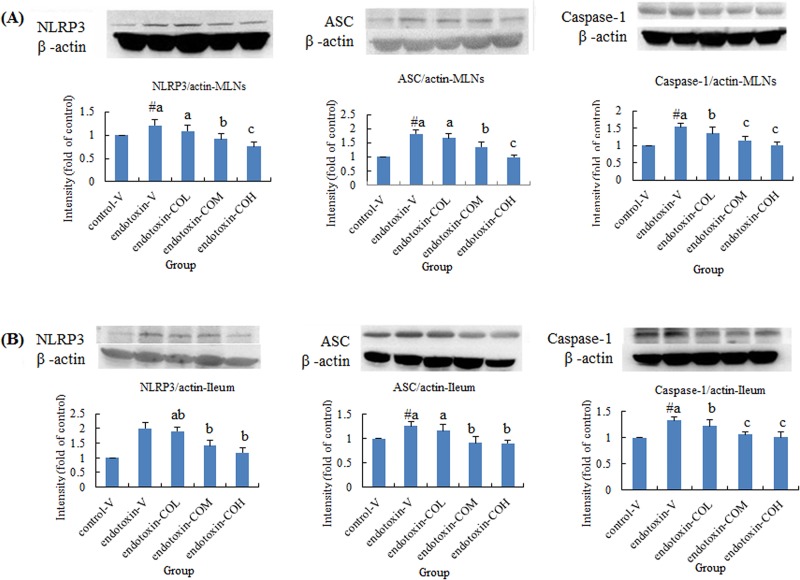
Effect of CO on the expression of NLRP3, ASC, and caspase-1 in mesenteric lymph nodes (A) and in ileum mucosa (B) of endotoxin-injected mice. Mice received by gavage 6.5 mg/kg CO (endotoxin-COL), 13 mg/kg CO (endotoxin-COM), 26 mg/kg CO (endotoxin-COH), or the vehicle (endotoxin-V) every other day 8 times followed by injection with endotoxin from *S*. *typhimurium* (i.p., 10 mg/kg body weight). Control mice were pretreated with vehicle (control-V) followed by injection with saline. Samples were collected at 12 h after injection. Data are means ± SDs for three mice in each group. #Significantly different from the control (p < 0.05). a, b, and c not sharing the same letter are significantly different (p < 0.05).

**Fig 4:**
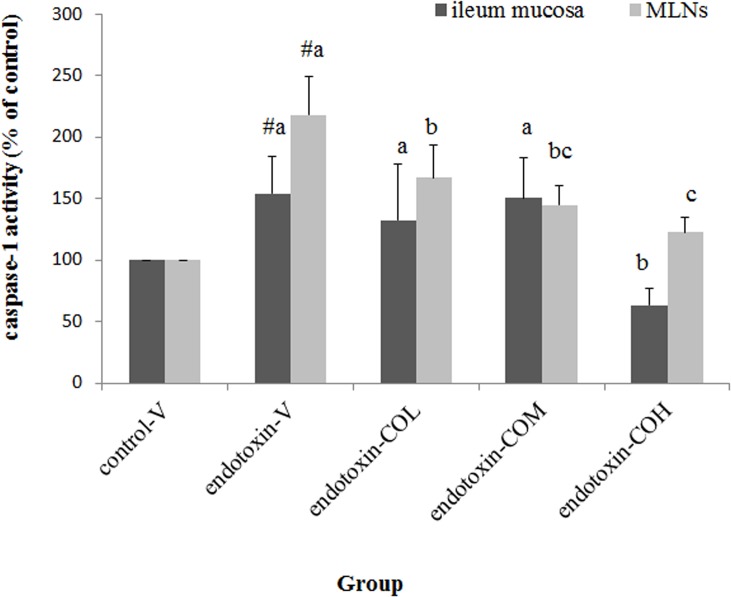
Effect of CO on caspase-1 activity in ileum mucosa and in mesenteric lymph nodes of endotoxin-injected mice. Mice received by gavage 6.5 mg/kg CO (endotoxin-COL), 13 mg/kg CO (endotoxin-COM), 26 mg/kg CO (endotoxin-COH), or the vehicle (endotoxin-V) every other day 8 times followed by injection with endotoxin from *S*. *typhimurium* (i.p., 10 mg/kg body weight). Control mice were pretreated with vehicle (control-V) followed by injection with saline. Samples were collected at 12 h after injection. Data are mean ± SD for six mice in each group. #Significantly different from the control (p < 0.05). a, b, and c the same tissue not sharing the same letter are significantly different (p < 0.05).

Induction of SIRS with endotoxin also raised the expression of NLRP3, ASC, and caspase-1 in both MLNs and ileum mucosa (Fig. 3A and B, respectively). In SIRS mice pretreated with CO, the expression of NLRP3, ASC, and caspase-1 in both tissues was significantly suppressed compared with that in the vehicle-treated mice, and this effect of CO was dose dependent (Fig. 3A and B). Further investigation of the total activity of caspase-1 in both tissues showed that the caspase-1 activity induced by endotoxin was significantly suppressed by CO. Particularly effective inhibitory activity was demonstrated in MLNs. The dose of 6.5 mg/kg CO showed significant inhibitory activity in MLNs. By contrast, in ileum mucosa, the only effective dose was 26 mg/kg CO (Fig. 4).

### Nitrate/Nitrite Content and Xanthine Oxidase Activity in Ileum Mucosa

**Fig 5:**
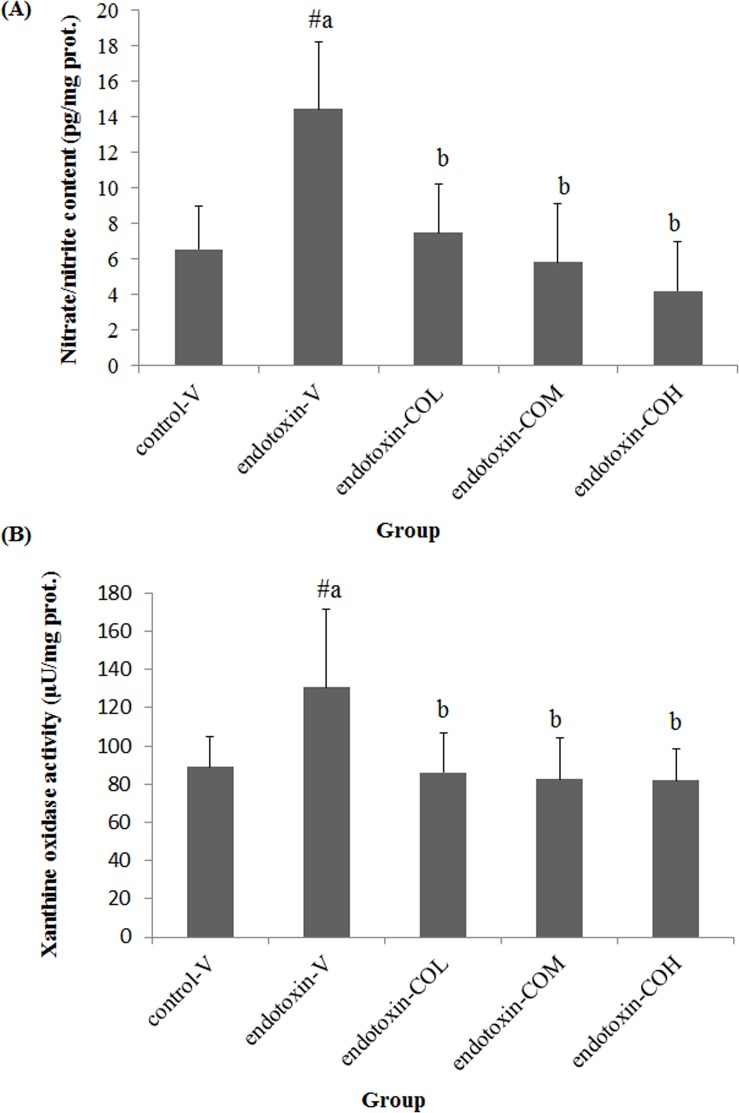
Effect of CO on nitrate/nitrite content (A) and xanthine oxidase activity (B) in ileum mucosa of endotoxin-injected mice. Mice received by gavage 6.5 mg/kg CO (endotoxin-COL), 13 mg/kg CO (endotoxin-COM), 26 mg/kg CO (endotoxin-COH), or vehicle (endotoxin-V) every other day 8 times followed by injection with endotoxin from *S*. *typhimurium* (i.p., 10 mg/kg body weight). Control mice were pretreated with vehicle (control-V) followed by injection with saline. Samples were collected 12 h after injection. Data are means ± SDs for six mice in each group. #Significantly different from the control (p < 0.05). a, b not sharing the same letter are significantly different (p < 0.05).

Similar to that in peripheral blood, endotoxin induced the elevation of nitrate/nitrite in ileum mucosa (Fig. 5A). Pretreatment with CO suppressed the induction of nitrate/nitrite in the ileum mucosa in a dose-dependent manner and by up to 48.2%, 59.6%, and 70.8% with 6.5, 13, and 26 mg/kg CO, respectively (Fig. 5A). We also found that SIRS led to increased xanthine oxidase activity in ileum mucosa (P<0.05), whereas pretreatment with all tested doses of CO effectively suppressed this activity (P<0.05, Fig. 5B).

### Histology of Ileum

**Fig 6:**
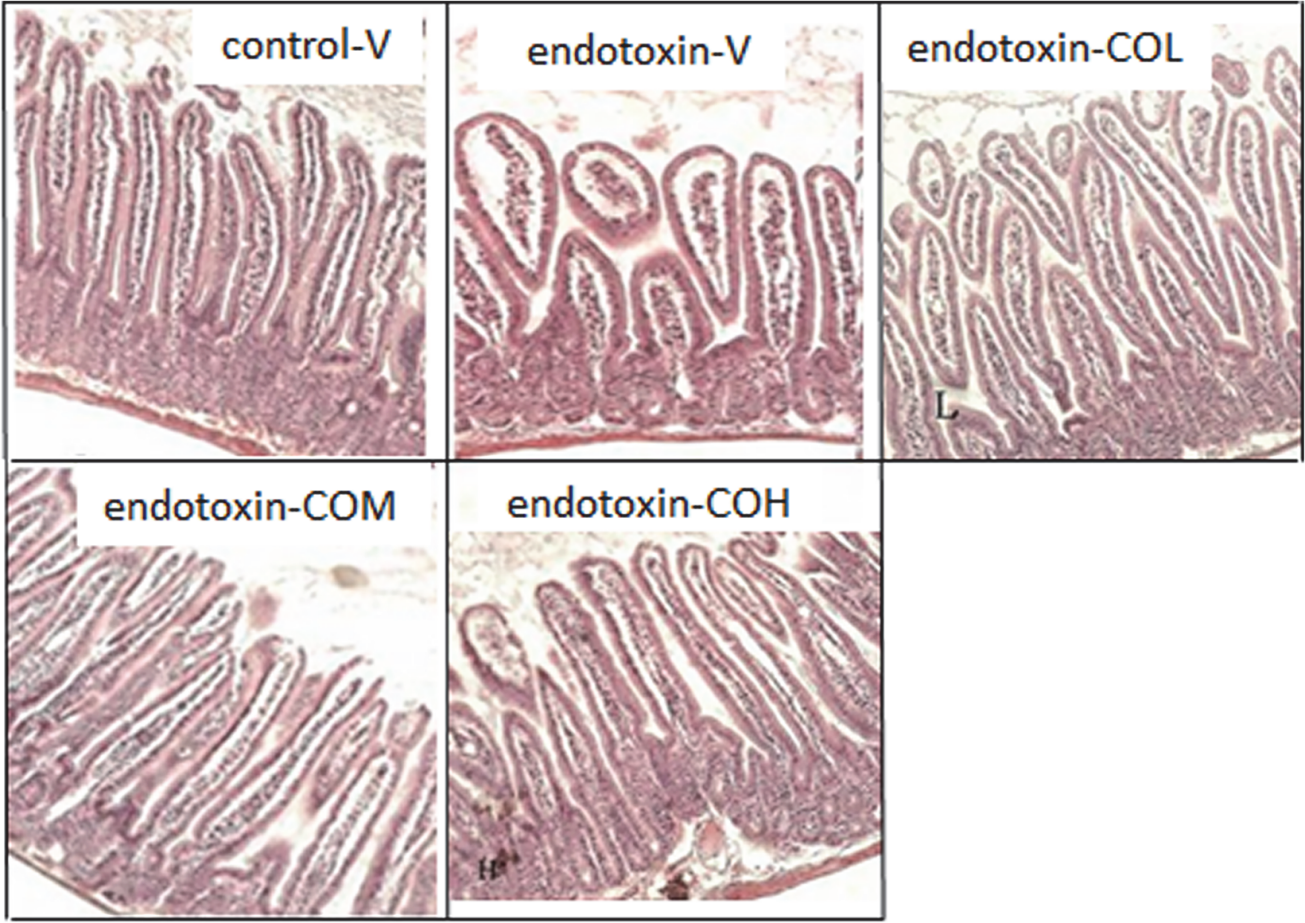
Cross-sections of ileum stained with hematoxylin and eosin. Mice received by gavage 6.5 mg/kg CO (endotoxin-COL), 13 mg/kg CO (endotoxin-COM), 26 mg/kg CO (endotoxin-COH), or the vehicle (endotoxin-V) every other day 8 times followed by injection with endotoxin from *S*. *typhimurium* (i.p., 10 mg/kg body weight). Control mice were pretreated with vehicle (control-V) followed by injection with saline. Samples were collected at 12 h after injection. Original magnification x 100.

The mucosal structure of the ileum was investigated by light microscopy. The morphology of the intestinal mucosa was impaired by the injection of endotoxin. In the vehicle-pretreated group, short, edematous villi with a flat top were observed (Fig. 6). Endotoxin-induced mucosal changes were much less severe in mice pretreated with CO; in these mice, the mucosal morphology was largely repaired and the height and sharp top of the villi were near normal, although the lamina propria remained slightly swollen (Fig. 6).

## Discussion and Conclusion

*Cinnamomum osmophloeum* Kanehira is an economic plant in Taiwan and has a long history of use by the natives of this island in food as a flavoring agent and in folk medicine for the relief of pain from acute arthritis, colds, stomachache, and gout. Being an indigenous plant of Taiwan, this plant has not gained much attention until recent years. Only 35 publications on this plant can be found in PubMed since 1986, and of these publications, 32 articles were published within the past 10 years. These reports show that this cinnamon species, especially its leaf essential oil (CO), possesses diverse bioactivity.

We previously reported the chemical composition of the CO of the linalool chemotype used in this study, which includes cinnamaldehyde (representing 6.87% of the CO composition) and linalool (representing 40.24% of the CO composition) as two potential compounds that possess anti-inflammatory activity [12]. Previous study showed that CO can inhibit the expression of pro-IL-1β and the production of IL-1β and IL-6 in endotoxin-induced J774A.1 macrophages [13]. By *in vitro* investigation with endotoxin-induced nitric oxide production in RAW 264.7 macrophages, the potent anti-inflammatory compositions in CO have been identified to include *trans*-cinnamaldehyde, T-cadinol, and α-cadinol [15]. Tung et al further reported that *trans*-cinnamaldehyde, (-)-aromadendrene, T-cadinol, and α-cadinol are the active compounds in CO responsible for anti-inflammatory effects *in vivo* in endotoxin/D-galactosamine-induced acute hepatitis [16]. According to our analysis, the CO used in this study composed very low amount of aromadendrene, T-cadinol, or α-cadinol representing 0.23%, 0.06%, and 0.03% of CO composition, respectively [12], therefore is unlikely to be the active compounds that provide the protective effect found in this study. The general constituent of cinnamon species, cinnamaldehyde, which is also a major active constituent in CO, has been shown to inhibit the endotoxin-induced production of IL-1β and TNF-α by J774A.1 macrophages and by THP-1 monocytes, to inhibit the TLR4 signaling pathway and to lower the intracellular level of pro-IL-1β in endotoxin-induced THP-1 monocytes [13], and to increase the level of two anti-inflammatory cytokines, IL-4 and IL-10, in OVA-injected mice *in vivo* [16]. As mentioned above, the CO used in this study composed 6.87% of cinnamaldehyde, this compound may play a role in the anti-inflammatory activity found in this study. Study on the anti-inflammatory activity of linalool rare. Recently, linalool was reported to inhibit endotoxin-induced activation of NF-κB and the production of TNF-α in RAW 264.7 macrophages and to attenuate endotoxin-induced lung injury in mice [18]. We proposed that the protective effect of CO on the endotoxin-induced systemic inflammatory response and intestinal damage found in the present study is largly provided by linalool. A comparison of the anti-inflammatory activity between cinnamaldehyde and linalool is ongoing in our lab at present.

It is well established that the elevated peripheral levels of proinflammatory cytokines IL-1β, TNF-α, and IFN-γ paly major role in the progressing of SIRS, and inhibition of the expression and the activity of IL-1β and TNF-α has become a therapeutic target for SIRS [22]. On the other hand, it has been known for years that peripheral concentrations of IL-18 are higher in septic patients than in healthy patients [23]. In experimental animals administered both lethal and nonlethal doses of endotoxin, IL-18 concentrations are dramatically elevated, and in the lethal model, the plasma level of IL-18 keeps increasing until the animal’s death [24]. In contrast, endotoxin-induced damage to the liver, heart, and lung in mice can be ameliorated with anti-IL-18 antibody or IL-18 binding protein [24–26]. The major role of IL-18 on systemic inflammation remained to be unclear. IL-18 was named an IFN-γ-inducer because of its known action on the induction of T-cells to produce IFN-γ, another proinflammatory cytokine attributed to tissue damage during sepsis [27]. IFN-γ can increase the expression of MD2 and TLR4 to amplify the consequences of endotoxin action [28]. A recent study demonstrated that IL-18 can mediate pro-apoptotic signaling in renal tubular cells [29], and a multicenter clinical study showed that in the case of survivors of critical illness complicated by acute kidney injury requiring renal replacement therapy, the plasma level of IL-18 is inversely associated with renal recovery and mortality [30]. How IL-18 would affect the intestinal cells and the associated complication remains to be clarified.

Consistent with these clinical and experimental observations, the present study demonstrated elevated peripheral levels of TNF-α, IL-1β, IL-18, and IFN-γ in endotoxin-injected mice, whereas we found that pretreatment with CO could suppress the levels of these cytokines in a dose-dependent manner. The suppressing effect of CO on peripheral levels of IL-1β and TNF-α is consistent with what was reported in an *in vitro* model [13, 14, 18]. However, the present study has reported for the first time that increased peripheral levels of IL-18 and IFN-γ in SIRS can be reduced by treatment with CO.

Gut barrier function under stress has been well accepted to be a major cause of damage to sites remote from the intestine and to play a major role in nosocomial infection in the critically ill condition [31]. The present study demonstrated that endotoxin injection induced intestinal damage in mice that was characterized by morphological changes such as edematous villi with a short height and flat top, which is consistent with what we reported in endotoxin-injected rats[19]. Although the present study did not determine the local content of proinflammatory cytokines in the intestine, with a similar SIRS model in rats, we previously reported that endotoxin elevated the intestinal content of IL-1β and TNF-α along with intestinal damage [32, 33].

In the present study, we directly addressed the underlying mechanisms of intestinal damage and have shown these to be associated with local activity of the TLR4 and NLRP3 pathways in the intestinal mucosa. On the other hand, failure of appropriate defense by the MLNs has also been proposed to be responsible for invasion of intestinal contents to peripheral blood, which initiates or worsens systemic injury. This proposed relation is referred to as the gut-lymph hypothesis [34]. Therefore, the present study also investigated changes in the local activity of the TLR4 and NLRP3 pathways in MLNs. We demonstrated, for the first time, that expression of the major components of the TLR4 and NLRP3 signaling pathways is induced in both intestinal mucosa and MLNs. Subsequently, the elevated activities of these pathways in both tissues were demonstrated by increased NF-κB activation and increased caspase-1 activity in endotoxin-induced SIRS.

Despite the role of cells in the innate immune system on the acute inflammatory condition, several lines of evidence suggest that TLR4 on epithelial cells may also be closely associated with the pathophysiology of endotoxin-induced intestinal mucosa injury. It has been shown that intestinal epithelial cells, including IEC-6 cells from rats, primary colonocytes, HT-29 and T84 colonocytes, and rectum CMT93 cells from mice, all express TLR4, MD2, and MyD88 [35]. *In vitro* study demonstrated that activation of TLR4 in enterocytes with endotoxin can promote apoptosis and blunt healing of the epithelium, which can deteriorate intestinal tissue damage and possibly promote bacterial translocation through this barrier [35]. The role of TLR4 in the inflammatory damage to intestinal epithelial cells is controversial. Given that mature epithelial cells constitutively express TLR4 and that the maintenance of normal barrier function may partly rely on the interaction between epithelial TLR4 and the commensal microbiota in the intestinal lumen, some researchers have proposed that deficiency in TLR4 signal transduction may disrupt the intestinal homoeostasis for protection from infectious injury [36]. The present study demonstrated that during endotoxin-induced SIRS, intestinal expression of molecules of the TLR4 signaling pathway and the activation of NF-κB, which is the downstream index of the activation of TLR4, are elevated in association with the development of mucosal damage. MLNs also showed the same trends in changes along with intestinal damage. We found that CO ameliorated this intestinal damage and that this effect is accompanied by suppressed expression of TLR4, MD2, and MyD88 and suppressed NF-κB activation reflecting by lowered level of this transcription factor in the nuclear fraction of the mucosal sample from mice received CO. It remains to be clarified whether the suppression of the TLR4 pathway by CO is a direct inhibitory effect or a result of ameliorated SIRS. In either case, however, the evidence for the anti-inflammatory activity of CO is solid with this model.

Although TLR4 has been the main target for the development of therapy for tissue damage in endotoxin-induced SIRS, there is increasing interest in NLRP3 [20]. Although endotoxin is not a ligand for the NLRP3 signaling pathway, a recent study demonstrated that NLRP3 expression can be induced by TLR agonists in murine macrophages in an NF-κB-dependent manner [37]. In addition, the expression of inducible nitric oxide synthase up-regulated by NF-κB can cause elevated production of nitric oxide and the subsequent hypotension and hypoxia that is responsible for the activation of local xanthine oxidase in tissues and organs [38]. Under such conditions, the NLRP3 inflammasome can be activated by uric acid generated from the activity of this enzyme [39, 40]. We previously reported that in endotoxin-induced intestinal damage, local inducible nitric oxide synthase activity and nitrate/nitrite content are significantly elevated [18]. The present study confirmed that increased activity of xanthine oxidase was induced in the intestine by endotoxin. It has been demonstrated that animals with NLRP3 or ASC deficiency can resist a lethal dose of endotoxin or septic shock, respectively [41, 42].

Although it has been shown that liver damage induced by endotoxin injection is associated with increased expression of local NLRP3, ASC, and caspase-1 and increased levels of IL-1β and IL-18 [20], the role of NLRP3 in intestinal damage induced by endotoxin has not been revealed. The present study found that upon induction with endotoxin, the activity of caspase-1 in both MLNs and in the intestinal mucosa was elevated, whereas this response was ameliorated by CO pretreatment. We interpreted this result as being at least partly through the suppression of the TLR4 pathway by CO and partly by the inhibitory effect of CO on the activity of xanthine oxidase. Nevertheless, the possibility that CO has a direct suppressive effect on the NLPR3 inflammasome cannot be excluded and remains to be clarified.

In conclusion, the present study for the first time provides strong evidence of the association of the TLR4 and NLRP3 signaling pathways with endotoxin-induced intestinal injury. This association was reflected by the increased local expression of TLR4, MD2, MyD88, NLRP3, ASC, and caspase-1 as well as by the increased local activation of NF-κB and the activity of caspase-1. In addition, this study for the first time showed an anti-inflammatory role of CO on endotoxin-induced systemic inflammation along with protective activity on intestinal damage. This effect of CO was inversely associated with the activity of local TLR4 and NLRP3 signaling pathways in the intestine. Thus, the results of the present study have shown that CO is a valuable natural product that has great potential for development as an anti-inflammation agent.
